# The analysis of reference genes expression stability in susceptible and resistant *Apera spica-venti* populations under herbicide treatment

**DOI:** 10.1038/s41598-021-01615-6

**Published:** 2021-11-12

**Authors:** Barbara Wrzesińska, Karolina Kościelniak, Patryk Frąckowiak, Tadeusz Praczyk, Aleksandra Obrępalska-Stęplowska

**Affiliations:** 1 Department of Molecular Biology and Biotechnology, Institute of Plant Protection – National Research Institute, Władysława Węgorka 20, 60-318 Poznan, Poland; 2 Department of Weed Science and Plant Protection Techniques, Institute of Plant Protection – National Research Institute, Władysława Węgorka 20, 60-318 Poznan, Poland

**Keywords:** Plant sciences, Plant molecular biology

## Abstract

Weed resistance to herbicides constitutes a serious problem to world crop production. One of the weeds that are significantly threatening the crops’ yield and quality is *Apera spica-venti*. The target-site resistance (TSR) mechanism of *A. spica-venti* has been widely studied, though, little is known about its non-target-site resistance (NTSR) mechanisms at the molecular level. Molecular examination of NTSR is, to a great extent, based on the expression profiles of selected genes, e.g. those participating in detoxification. However, to obtain reliable results of gene expression analysis, the use of a normalizer is required. The aim of this study was to select the best reference genes in *A. spica-venti* plants of both populations, susceptible and resistant to ALS inhibitor, under treatment with herbicide. Eleven housekeeping genes were chosen for their expression stability assessment. The efficiency correction of raw quantification cycles (Cq) was included in the gene expression stability analyses, which resulted in indicating the TATA-box binding protein (*TBP*), glyceraldehyde-3-phosphate dehydrogenase, cytosolic (*GAPC*), and peptidyl-prolyl *cis–trans* isomerase CYP28 (*CYP28*) genes as the most stably expressed reference genes. The obtained results are of vital importance for future studies on the expression of genes associated with the non-target-site resistance mechanisms in the *A. spica-venti* populations susceptible and resistant to herbicides.

## Introduction

Herbicide resistance is one of the most problematic issues in agriculture. Repeated applications of herbicides in fields created the selection pressure that resulted in the emergence of herbicide-resistant weed populations. One of the commonly occurring weeds belonging to the Poaceae family is silky windgrass (*Apera spica-venti* L.). It occurs in many countries of central and northern Europe and endangers winter cereal crops, in particular^1,2^. Its spread potential is explained by the adaptation capabilities to inhabit numerous farming environments^3,4^. Silky windgrass populations can easily develop herbicide resistance due to such traits as widespread distribution, cross-pollination, high seed production, and low primary dormancy^5^. One plant can produce up to 16,000 seeds, which are dispersed by water and wind^6^. *A. spica-venti* has been controlled using acetolactate synthase (ALS) and acetyl-CoA (ACCase) inhibitors, as well as, to a lesser extent, photosystem II inhibitors^2^. However, their long-term use in fields has brought about development of *A. spica-venti* resistance to them.

There are two mechanisms of herbicide resistance, the target-site resistance (TSR) and non-target-site resistance (NTSR). TSR is mainly caused by mutations occurring at the site of herbicide action in the target enzyme, decreasing the affinity of the herbicide active substance. Additionally, less frequently TSR may result from increased expression of the gene being the herbicide target^7^. Currently, in silky windgrass, seven target-site mutations at three positions in the ALS gene are known^5^. NTSR mechanism encompasses decreased rates of herbicide uptake/translocation/penetration/activation, or increased herbicide metabolism/sequestration^7^. NTSR mechanism involves the induction of expression of a wide variety of genes and gene families, such as encoding cytochrome P450 monooxygenases (*CYP450s*), ABC transporters (*ABC*), glutathione S-transferases (*GST*), glycosyltransferases (*GT*), esterases, and oxidases^8^. The elevated levels of CYP450s, GT, and GST typically contribute to herbicide detoxification, which is divided in three phases. In the first phase, often mediated by CYP450s, a functional group is added to the herbicide molecule converting it to a more hydrophilic metabolite. Phase II is associated with the conjugation to GSH (mediated by GST) or to glucose (mediated by GT). Finally, in phase III, herbicide metabolites are exported to the vacuole or incorporated into cell wall^8,9^.

The involvement of the mentioned gene families in the NTSR may be studied by the gene expression level analysis. There are several methods to study changes in gene expression, including the quantitative reverse transcription polymerase chain reaction (RT-qPCR), the next-generation sequencing (RNAseq), microarrays, and Northern blotting. RT-qPCR is the most popular and the most commonly used method for the assessment of genes expression level. It is also frequently used for the validation of results obtained in high-throughput analyses. However, to perform a reliable RT-qPCR analysis devoid of the technical and sample variations, a normalization of the expression data using a reference gene or genes is required. The most stably expressed genes should be identified for each type of conditions individually. Both, biotic and abiotic stresses acting on different plant species influence the stability of expression of the potential reference genes and thus the reference gene choice. The studies on reference gene expression stability in weeds belonging to the Poaceae family, *Alopecurus myosuroides* and *Eleusine indica,* exposed to herbicide stress have revealed that in the plants subjected to the same stress, different genes exhibited the most stable expressions. Ubiquitin-40S ribosomal protein (*UBQ*), tubulin (*TUB*), and glyceraldehyde-3-phosphate dehydrogenase (*GAPDH*) were the most stably expressed genes in *A. myosuroides*, while ubiquitin-conjugating enzyme (*UBC*), eukaryotic initiation factor 4A, and elongation factor 1-alpha (*EF1A*) were indicated as the most stably expressed in *E. indica*^10,11^. Moreover, *TUB* was identified to show unstable expression in *E. indica*.

Usually, reference genes used in expression analyses are chosen from those constitutively expressed in all cell types (housekeeping genes, HKGs) because they are required for cell survival^12^. Identification of the most stably expressed genes in the experiments concerning herbicide resistance in different systems is also crucial. Identification of reference genes under treatment with herbicide has been the subject of a number of studies on weed species such as *A. myosuroides*^10^, *E. indica*^11^, *Lolium rigidum*^13^, or *Avena fatua*^14^. However, no reference gene expression stability analysis has been performed on the herbicide-resistant and susceptible populations of *A. spica-venti*. Moreover, the issue concerning primer efficiency correction of raw quantification cycles (Cq) subjected to statistical analyses of gene expression stability has been usually discounted. The consequences of taking into account the raw Cq values in gene expression stability analyses may result in discrepancies in indicating the most stably expressed genes, relative to the results obtained from analyses of the efficiency corrected data^15^. Therefore, in this study 11 HKGs were chosen for the expression stability analysis in *A. spica-venti*: actin (*ACT*), ADP-ribosylation factor 1 (*ARF1*), peptidyl-prolyl *cis–trans* isomerase CYP28 (*CYP28*), *EF1A*, *GAPC*, 60S ribosomal protein L23a (*RPL23A*), Rubisco activase (*RCA*), sucrose-phosphate synthase (*SPS*), TATA-box binding protein (*TBP*), *UBC*, and *UBQ*. The aim of this study was to determine the best reference genes for determination of selected genes’ expression stability profiles in susceptible and resistant *A. spica-venti* plants exposed or not to treatment with herbicide at two time points (1 h and 24 h). For statistical analysis of the gene expression stability, two series of input data were used: efficiency corrected and non-efficiency corrected Cq values. Additionally, the most stably expressed reference genes in susceptible or resistant plants were also identified.

By employing RT-qPCR and four statistical algorithms for analysis of the efficiency corrected Cq values, the genes: *TBP*, *GAPC*, and *CYP28* were found among the most stably expressed HKGs in all tested samples, while *CYP28*, *SPS*, and *GAPC*, and, *TBP*, *GAPC*, and *ACT* had the highest expression stability values in susceptible and resistant plants, respectively. Moreover, it was shown that two reference genes were sufficient for the accurate normalization of RT-qPCR results. At the final point of the study, the validation of the *ABCC10* and *CYP89A2* genes expression, with the use of *TBP* and *GAPC*, revealed the involvement of these detoxification-related genes in *A. spica-venti* response to a herbicide belonging to the sulfonamide group.

## Results

### Plant populations selection for analysis of the expression stability of housekeeping genes

Eleven populations (two susceptible and nine resistant) were examined in order to determine ED50 (effective dose of active ingredient (ai) causing a 50% reduction in plant biomass). In the susceptible populations, ED50 was lower than 1/16N dose (N—the maximal recommended dose of the herbicide (120 g ha^−1^, i.e. 9 g ha^−1^ of ai)) (Table S1). For four out of the nine resistant populations the ED50 values were higher than 32N dose of the herbicide. In order to choose *A. spica-venti* populations to be subjected to HKGs stability analysis, plants from the populations resistant and susceptible to pyroxsulam were examined for the absence of mutations in the *ALS* sequence. *ALS* sequence analysis permitted selection of two susceptible and two resistant *A. spica-venti* populations without known mutations conferring the resistance. Single mutations were found, however, they were present in both susceptible and resistant plants, or the interdomain region of ALS.

### RT-qPCR primer specificity and efficiency

**Figure 1:**
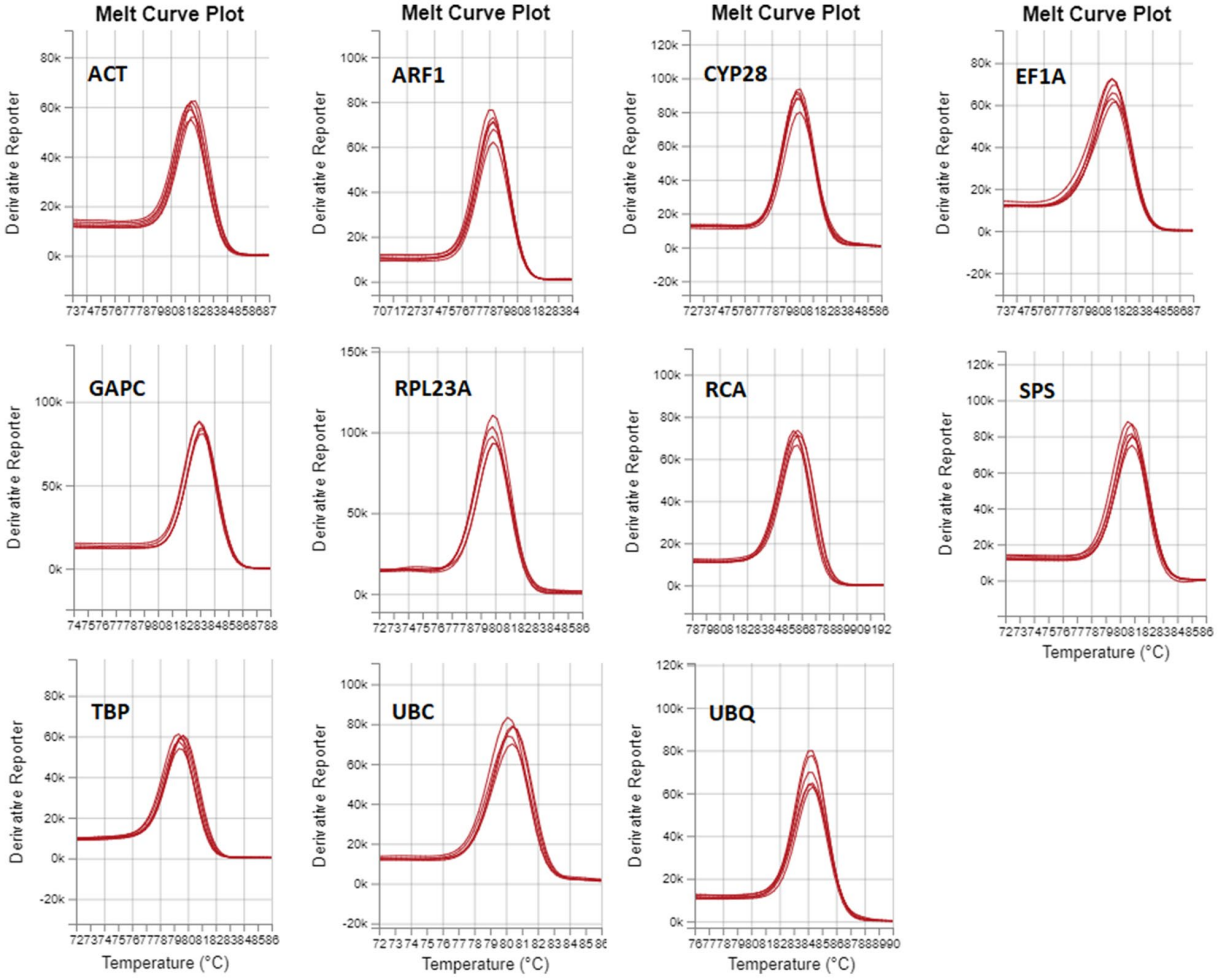
Melting curve plots for candidate reference genes.

**Figure 2:**
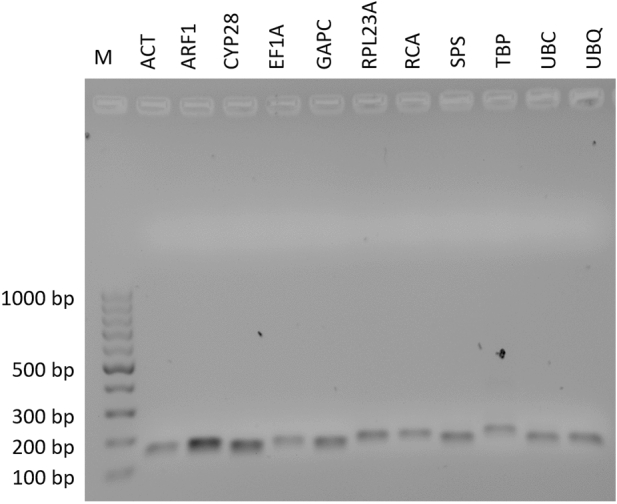
RT-qPCR amplification products of *A. spica-venti* candidate reference genes. *M* DNA ladder, *bp* base pairs.

In order to choose the most reliable reference genes in susceptible and resistant to ALS inhibitors plants of *A. spica-venti* exposed to treatment with herbicide, 11 candidate HKGs were selected for the expression stability analysis. These candidates were the primary metabolism genes frequently used as reference ones in other weeds species and were indicated as stable in other plants exposed to different abiotic stresses. The specificity of the primers used was confirmed by a single peak of melting curves (Fig. 1) and agarose gel electrophoresis (Fig. 2). The sequences of RT-qPCR products were compared to the previously published *A. spica-venti* transcriptome data (NCBI Gene Expression Omnibus GSE86989)^16^ showing high identity to the corresponding genes in the transcriptome. These sequences were deposited in the GenBank database (accession numbers provided in Table 1). To confirm the similarity of the obtained sequences to the selected genes, the latter were blasted in the following databases: NCBI blastn and UniProt blastp, as well as, against model plant species *Arabidopsis thaliana* and *Brachypodium distachyon*. The results with the highest scores are presented in Table S2.

**Table 1:** Primer sequences for RT‐qPCR used in this study.

Analysis	Gene	Accession numbers of the sequences in *A. spica-venti* transcriptome/sequenced in this study and deposited in GenBank	Primer sequence (5′–3′)	Amplicon length (bp)	Annealing temperature (°C)	RSq	PCR efficiency (%)
Reference genes expression stability analysis	ACT	TRINITY_DN132310_c0_g1_i1|m.143952	F: CGCTTATGTTGCCCTTGATT	151	64	0.994	91.4
		MW712586	R: AAGAGATGGCTGGAAAAGCA				
	ARF1	TRINITY_DN237260_c0_g1_i2|m.37134	F: GTTCCAACTGTGGGGCTTAAT	157	60	0.998	94.8
		MW712587	R: ATGCAGAGGCAGAGTCGATAA				
	CYP28	TRINITY_DN244654_c2_g6_i1|m.54263	F: GTCCACCTCCACAATTGACAC	146	62	0.997	99.9
		MW712588	R: CAAAGACTACACGGCCAAGAG				
	EF1A	TRINITY_DN251778_c2_g5_i3|m.44674	F: CCGAGCGTGAGAGAGGTATC	157	56	0.998	108.2
		MW712589	R: TCAATGATGAGCACAGCACA				
	GAPC	TRINITY_DN238590_c0_g1_i2|m.43942	F: CAGTCACTGTCTTCGGTGTCA	151	58	0.999	106.7
		MW712590	R: GCAGAGATGACCACCTTCTTG				
	RPL23A	TRINITY_DN242121_c1_g10_i1|m.107926	F: AAGTACCCCAGGATCAGCACT	166	60	0.999	88.2
		MW712591	R: TGACAGCAGCCTTGATCTTCT				
	RCA	TRINITY_DN254390_c0_g3_i5|m.45789	F: ACTACCATGGCAAGAGCTTCA	167	54	0.999	105.6
		MW712592	R: GAAGAGGGAGTCCACGATAC				
	SPS	TRINITY_DN256108_c2_g8_i1|m.2492	F: CATGCAGATGTCCAAGGTTCT	152	62	0.997	99.8
		MW712593	R: GAGAATGGCCTGTGAATACCA				
	TBP	TRINITY_DN243731_c0_g18_i1|m.150479	F: TCGTTGGCTCTTGTGATGTC	175	64	0.999	90.5
		MW712594	R: TTTGCTCCGGTCAAGACAAT				
	UBC	TRINITY_DN257614_c2_g3_i2|m.4201	F: TGGTGCATGTGAACTGGATAA	149	60	0.998	92.6
		MW712595	R: AGACAGAGTGCACCAATCACC				
	UBQ	TRINITY_DN236450_c0_g17_i1|m.156536	F: CAACATCCAGAAGGAGTCCAC	156	60	0.998	98.6
		MW712596	R: CCGTCGTCGACCTTATAGAAC				
Validation	ABCC10	TRINITY_DN231466_c1_g3_i1|m.91733	F: TCTTGGTGTGGCGTTTGTTC	113	60	0.998	113.4
		MW712597	R: CCTTGATGGTGATCGTCTGCT				
	CYP82A2	TRINITY_DN252745_c0_g2_i2|m.101768	F: GGACACTCTGCTCGACATCA	170	60	0.998	103.0
		MW712598	R: AGAGCTTGTGCTGGATGGAT				

*RSq* R‐squared value for standard curve.

**Figure 3:**
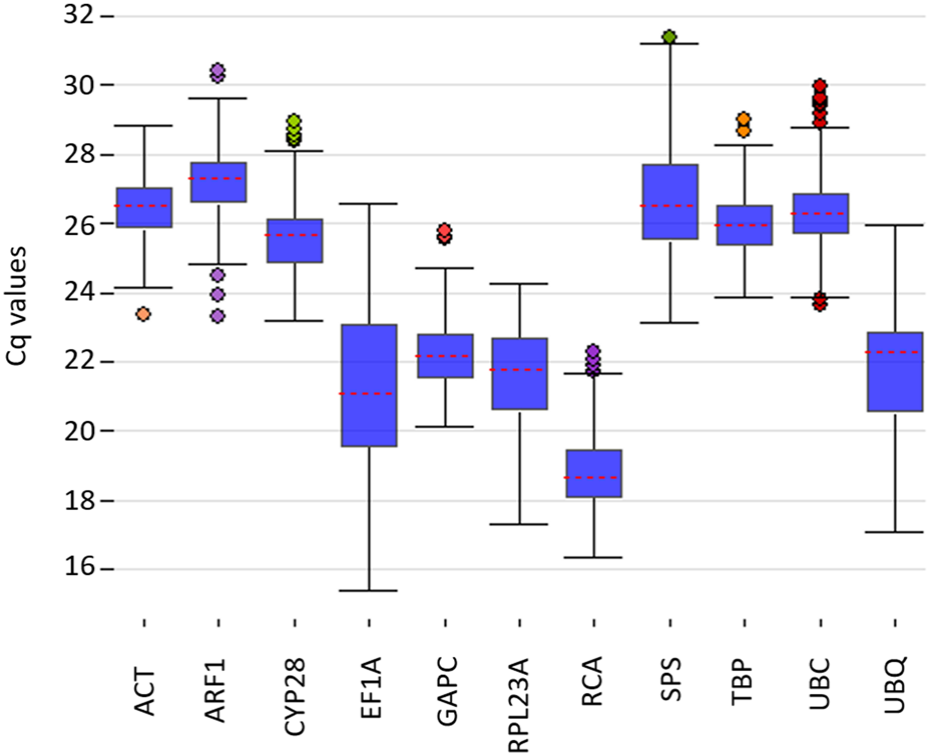
Distribution of the raw RT-qPCR quantification cycle (Cq) values for the candidate reference genes.

The analysis of standard curves resulted in the amplification efficiency ranging from 88.2% (*RPL23A*) to 108.2% (*EF1A*), while the regression coefficient varied from 0.994 (*ACT*) to 0.999 (*GAPC*, *RPL23A*, *RCA*, and *TBP*) (Table 1). The distribution of Cq values revealed the highest expression of *RCA* with the mean Cq value of 18.82, while the lowest mean Cq value was observed for *ARF1* (27.19) (Fig. 3). The lowest Cq range (5.11 cycles) was detected for the *TBP*, whereas the greatest discrepancy in Cq values (11.15) was observed for *EF1A*.

### Analysis of the candidate reference genes expression stability

The HKGs expression stability analyses were performed with the following datasets: the first one consisted of data taken from all samples, the second one—the data obtained for the samples from susceptible plants, and the third one—the data collected for the samples from resistant plants. Four statistical tools were used to conduct the analysis: geNorm^17^, BestKeeper^18^, NormFinder^19^, and ΔC_t_^20^. To gain a comprehensive summary of the results, RefFinder software (https://www.heartcure.com.au/reffinder) was used.

Most of the gene expression stability analysis softwares omit the fact that not every primer pair and reaction conditions ensure 100% efficiency in RT-qPCR. The use of qPCR efficiency corrected Cq values in statistical analyses of the most stably expressed genes impacts the final expression stability ranking, which may result in selection of different sets of reference genes for normalization. Therefore, the efficiency correction was applied to the raw Cq values.

#### Candidate reference genes expression stability analysis in all samples indicated that *TBP*, *GAPC*, and *CYP28* show the most stable expression under tested conditions

The first analysis, performed with the geNorm software, using Bioconductor “NormqPCR” package in R software, indicated that the first three of the most stably expressed genes were *TBP*, *ARF1*, and *GAPC*, while the genes with the least stable expression were—*EF1A*, *UBQ*, and *RPL23A* (Table 2). The last three genes also exhibited unstable expression according to BestKeeper and NormFinder analyses. The genes found to be the most stably expressed by BestKeeper were *TBP*, *ACT*, and *GAPC*, whereas the ones *CYP28*, *TBP*, and *GAPC* were indicated as the most stably expressed by NormFinder. Similarly, the ΔCt method pinpointed *CYP28*, *TBP*, and *GAPC* to have the highest expression stability, contrary to *RCA, EF1A*, and *RPL23A* that were classified as the least stably expressed. The general ranking obtained from RefFinder analysis indicated *TBP*, *GAPC*, and *CYP28* as the reference genes with the most stable expression in the plants susceptible and resistant to pyroxsulam, and *EF1A*, *UBQ*, and *RPL23A* as the least stably expressed ones.

**Table 2:** Expression stability ranking of eleven candidate genes in *Apera spica-venti* plants resistant and susceptible to pyroxsulam.

Analysis	Sample data	Genes expression stability										
		Most stable					Less stable					
GeNorm (M value)	All samples	*TBP*	*ARF1*	*GAPC*	*ACT*	*CYP28*	*UBC*	*SPS*	*RCA*	*RPL23A*	*UBQ*	*EF1A*
		0.044	0.044	0.044	0.046	0.050	0.053	0.056	0.059	0.070	0.076	0.087
	Susceptible	*CYP28*	*SPS*	*TBP*	*ACT*	*GAPC*	*ARF1*	*UBC*	*RCA*	*RPL23A*	*UBQ*	*EF1A*
		0.034	0.034	0.042	0.044	0.046	0.049	0.052	0.056	0.070	0.078	0.086
	Resistant	*TBP*	*ARF1*	*ACT*	*GAPC*	*CYP28*	*UBC*	*SPS*	*RCA*	*UBQ*	*RPL23A*	*EF1A*
		0.034	0.034	0.034	0.035	0.038	0.041	0.045	0.048	0.053	0.058	0.068
BestKeeper (SD)	All samples	*TBP*	*ACT*	*GAPC*	*ARF1*	*CYP28*	*RCA*	*UBC*	*SPS*	*RPL23A*	*UBQ*	*EF1A*
		0.705	0.795	0.801	0.869	0.894	0.926	0.955	1.220	1.338	1.409	1.918
	Susceptible	*TBP*	*ACT*	*SPS*	*GAPC*	*RCA*	*CYP28*	*ARF1*	*UBC*	*RPL23A*	*UBQ*	*EF1A*
		0.694	0.716	0.847	0.878	0.907	0.926	0.982	1.063	1.617	1.653	1.874
	Resistant	*GAPC*	*ACT*	*TBP*	*ARF1*	*CYP28*	*RCA*	*UBC*	*UBQ*	*SPS*	*RPL23A*	*EF1A*
		0.690	0.698	0.716	0.736	0.777	0.790	0.870	1.038	1.049	1.062	1.526
NormFinder (SD)	All samples	*CYP28*	*TBP*	*GAPC*	*ARF1*	*RCA*	*ACT*	*SPS*	*UBC*	*RPL23A*	*UBQ*	*EF1A*
		0.530	0.578	0.617	0.650	0.899	0.936	0.996	1.015	1.120	1.219	1.775
	Susceptible	*CYP28*	*SPS*	*GAPC*	*TBP*	*ARF1*	*ACT*	*RCA*	*UBQ*	*RPL23A*	*UBC*	*EF1A*
		0.438	0.558	0.682	0.685	0.745	0.788	0.967	1.250	1.252	1.255	1.625
	Resistant	*GAPC*	*TBP*	*CYP28*	*ARF1*	*ACT*	*UBQ*	*UBC*	*RCA*	*RPL23A*	*SPS*	*EF1A*
		0.350	0.402	0.529	0.555	0.684	0.696	0.740	0.753	0.940	0.948	1.305
ΔCT (mean SD)	All samples	*CYP28*	*TBP*	*GAPC*	*UBC*	*ACT*	*SPS*	*UBQ*	*ARF1*	*RPL23A*	*EF1A*	*RCA*
		1.683	1.711	1.878	1.896	1.910	1.991	2.056	2.079	2.094	2.439	3.044
	Susceptible	*TBP*	*CYP28*	*ACT*	*UBC*	*GAPC*	*EF1A*	*ARF1*	*SPS*	*RPL23A*	*UBQ*	*RCA*
		1.460	1.467	1.500	1.670	1.700	1.772	1.800	1.804	1.840	1.865	2.622
	Resistant	*CYP28*	*TBP*	*GAPC*	*SPS*	*UBC*	*UBQ*	*ACT*	*RPL23A*	*ARF1*	*EF1A*	*RCA*
		1.388	1.458	1.516	1.525	1.529	1.545	1.700	1.723	1.814	2.217	2.813
RefFinder (Geomean)	All samples	*TBP*	*GAPC*	*CYP28*	*ARF1*	*ACT*	*RCA*	*UBC*	*SPS*	*RPL23A*	*UBQ*	*EF1A*
		1.190	2.280	2.510	3.720	4.740	5.230	6.960	7.740	9.000	10.000	11.000
	Susceptible	*CYP28*	*SPS*	*TBP*	*GAPC*	*ACT*	*ARF1*	*RCA*	*UBC*	*UBQ*	*RPL23A*	*EF1A*
		1.570	1.860	2.830	3.220	4.160	6.190	6.190	8.460	8.970	9.490	11.000
	Resistant	*GAPC*	*TBP*	*ARF1*	*ACT*	*CYP28*	*UBC*	*RCA*	*UBQ*	*SPS*	*RPL23A*	*EF1A*
		1.000	2.450	2.830	3.760	3.870	6.480	6.960	7.670	8.970	9.740	11.000

The input data for the geNorm, BestKeeper, ΔC_t_, and RefFinder calculations were the raw Cq values.

#### Analysis of the reference genes expression stability separately for the pyroxsulam susceptible and resistant plants indicated that *TBP* was among the most stably expressed genes in both types of samples

The same methods were used in the analyses of HKGs expression stability, separately in the plants showing the susceptibility or resistance to sulfonamide herbicides.

In the susceptible plants (Table 2), the genes exhibiting the highest expression stability were as follows: *CYP28*, *SPS*, and *TBP* (according to geNorm); *TBP*, *ACT*, and *SPS* (according to BestKeeper); *CYP28*, *SPS*, and *GAPC* (according to NormFinder); *TBP*, *CYP28*, and *ACT* (according to ΔCt method). The least stably expressed genes were: *EF1A*, *UBQ,* and *RPL23A* (according to geNorm and BestKeeper); *EF1A*, *UBC*, and *RPL23A* (according to NormFinder); *RCA*, *UBQ*, and *RPL23A* (according to ΔCt method). The comprehensive ranking established using RefFinder showed that *CYP28*, *SPS,* and *TBP* were characterized by the highest expression stability values, and *EF1A*, *RPL23A*, and *UBQ* were indicated to have unstable expression.

In the resistant plants (Table 2), the genes classified to be the most stably expressed were as follows: *TBP*, *ARF1*, and *ACT* (according to geNorm); *GAPC*, *ACT*, and *TBP* (according to BestKeeper); *GAPC, TBP*, and *CYP28* (according to NormFinder); *CYP28*, *TBP*, and *GAPC* (according to ΔCt method). The genes identified as the least stably expressed were: *EF1A*, *RPL23A*, and *UBQ* (according to geNorm); *EF1A*, *RPL23A*, and *SPS* (according to BestKeeper); *EF1A*, *SPS*, and *RPL23A* (according to NormFinder); *RCA*, *EF1A*, and *ARF1* (according to ΔCt method). RefFinder analysis indicated *GAPC*, *TBP*, and *ARF1* as the best reference genes with the most stable expression, while *EF1A*, *RPL23A*, and *SPS* showed the least stability in their expression.

#### Statistical analyses of efficiency adjusted Cq values has changed the positions of the candidate reference genes in the expression stability ranking

The efficiency correction to the raw Cq values obtained from RT-qPCR was applied using the GenEx software. The values of efficiency used are given in Table 1. The gene expression stability analysis using the corrected Cq values was performed with all statistical methods used in the previous analyses namely: geNorm, BestKeeper, NormFinder, ΔCt, and RefFinder. This approach resulted in differences in the gene expression stability ranking (Table 3). Then, two genes expression stability rankings based on the data resulting from analyses of efficiency corrected Cq and non-corrected Cq were compared. No differences in the results obtained from geNorm analyses were observed. The results of the BestKeeper and NormFinder softwares revealed the changes in 39% (13/33) and 33% (11/33) of HKGs positions, respectively. The biggest discrepancies in the rankings were observed in the results obtained by the ΔCt method, with 73% (24/33) of changes in genes positions. The comprehensive ranking obtained by RefFinder showed that 45% (15/33) HKGs positions were altered.

**Table 3:** Expression stability ranking of eleven candidate genes in *Apera spica-venti* plants resistant and susceptible to pyroxsulam.

Analysis	Sample data	Genes expression stability										
		Most stable					Less stable					
geNORM (M value)	All samples	*ARF1*	*TBP*	*GAPC*	*ACT*	*CYP28*	*UBC*	*SPS*	*RCA*	*RPL23A*	*UBQ*	*EF1A*
		0.044	0.044	0.044	0.046	0.050	0.053	0.056	0.059	0.070	0.076	0.087
	Susceptible	*CYP28*	*SPS*	*TBP*	*ACT*	*GAPC*	*ARF1*	*UBC*	*RCA*	*RPL23A*	*UBQ*	*EF1A*
		0.034	0.034	0.042	0.044	0.046	0.049	0.052	0.056	0.070	0.078	0.086
	Resistant	*TBP*	*ARF1*	*ACT*	*GAPC*	*CYP28*	*UBC*	*SPS*	*RCA*	*UBQ*	*RPL23A*	*EF1A*
		0.034	0.034	0.034	0.035	0.038	0.041	0.045	0.048	0.053	0.058	0.068
BestKeeper (SD)	All samples	*TBP*	*ACT*	*ARF1*	*GAPC*	*CYP28*	*UBC*	*RCA*	*SPS*	*RPL23A*	*UBQ*	*EF1A*
		0.655	0.744	0.836	0.839	0.893	0.903	0.963	1.218	1.221	1.395	2.029
	Susceptible	*TBP*	*ACT*	*SPS*	*GAPC*	*CYP28*	*RCA*	*ARF1*	*UBC*	*RPL23A*	*UBQ*	*EF1A*
		0.645	0.671	0.846	0.920	0.926	0.943	0.945	1.005	1.475	1.637	1.982
	Resistant	*ACT*	*TBP*	*ARF1*	*GAPC*	*CYP28*	*RCA*	*UBC*	*RPL23A*	*UBQ*	*SPS*	*EF1A*
		0.653	0.665	0.708	0.722	0.777	0.822	0.822	0.969	1.028	1.047	1.614
NormFinder (SD)	All samples	*CYP28*	*TBP*	*ARF1*	*GAPC*	*ACT*	*RCA*	*UBC*	*SPS*	*RPL23A*	*UBQ*	*EF1A*
		0.520	0.576	0.631	0.634	0.913	0.919	0.967	0.987	1.004	1.213	1.920
	Susceptible	*CYP28*	*SPS*	*TBP*	*GAPC*	*ARF1*	*ACT*	*RCA*	*RPL23A*	*UBC*	*UBQ*	*EF1A*
		0.435	0.553	0.680	0.704	0.715	0.774	0.984	1.104	1.199	1.239	1.768
	Resistant	*GAPC*	*TBP*	*CYP28*	*ARF1*	*ACT*	*UBQ*	*UBC*	*RCA*	*RPL23A*	*SPS*	*EF1A*
		0.341	0.395	0.522	0.547	0.668	0.688	0.703	0.772	0.854	0.946	1.426
ΔCT (mean SD)	All samples	*TBP*	*GAPC*	*ACT*	*UBC*	*CYP28*	*ARF1*	*UBQ*	*SPS*	*EF1A*	*RPL23A*	*RCA*
		1.421	1.475	1.563	1.593	1.641	1.827	1.917	2.055	2.090	2.476	2.492
	Susceptible	*TBP*	*ACT*	*GAPC*	*UBC*	*CYP28*	*EF1A*	*ARF1*	*UBQ*	*SPS*	*RCA*	*RPL23A*
		1.238	1.266	1.315	1.415	1.497	1.556	1.586	1.790	1.960	2.115	2.288
	Resistant	*TBP*	*GAPC*	*UBC*	*ACT*	*CYP28*	*UBQ*	*SPS*	*ARF1*	*EF1A*	*RPL23A*	*RCA*
		1.118	1.137	1.202	1.262	1.266	1.353	1.515	1.551	1.656	2.052	2.250
RefFinder (Geomean)	All samples	*TBP*	*CYP28*	*GAPC*	*ARF1*	*ACT*	*RCA*	*UBC*	*SPS*	*RPL23A*	*UBQ*	*EF1A*
		1.410	1.860	2.280	4.680	4.700	5.730	5.790	7.740	9.000	10.000	11.000
	Susceptible	*CYP28*	*SPS*	*GAPC*	*TBP*	*ACT*	*RCA*	*ARF1*	*UBC*	*RPL23A*	*UBQ*	*EF1A*
		1.500	1.860	3.220	3.360	3.980	6.190	6.320	7.420	8.740	10.000	11.000
	Resistant	*TBP*	*GAPC*	*ACT*	*CYP28*	*ARF1*	*UBC*	*RCA*	*UBQ*	*RPL23A*	*SPS*	*EF1A*
		1.190	1.860	2.940	3.870	4.000	6.480	7.200	7.640	8.970	9.460	11.000

The input data consisting for geNorm, BestKeeper, NormFinder, ΔCt, and RefFinder calculations of were Cq values after efficiency correction.

In all samples analyzed (Table 3), the three most stably expressed genes taking into account the efficiency correction were as follows: *ARF1*, *TBP*, and *GAPC* (according to geNorm); *TBP*, *ACT*, and *ARF1* (according to BestKeeper); *CYP28*, *TBP*, and *ARF1* (according to NormFinder); *TBP*, *GAPC*, and *ACT* (according to ΔCt method); *TBP*, *CYP28*, and *GAPC* (according to RefFinder). The ranking of the least stably expressed genes changed only according to the results of ΔCt method and comprised *RCA*, *RPL3A*, and *EF1A.*

In the samples collected from the susceptible plants, the three most stably expressed genes taking into account the efficiency correction were as follows: *CYP28*, *SPS*, and *TBP* (according to geNorm); *TBP*, *ACT*, and *SPS* (according to BestKeeper); *CYP28*, *SPS*, and *TBP* (according to NormFinder); *TBP*, *ACT*, and *GAPC* (according to ΔCt method); *CYP28*, *SPS*, and *GAPC* (according to RefFinder). Changes in the order of the unstably expressed genes were found in the results obtained from NormFinder (for genes *EF1A*, *UBQ*, and *UBC*), ΔCt (for *RPL23A*, *RCA*, and *SPS*) and RefFinder (for *EF1A*, *UBQ*, and *RPL23A*).

The greatest differences in the initial three positions were found in the results obtained for the samples harvested from the resistant plants. The most stably expressed genes after efficiency correction ranked as follows: *TBP*, *ARF1*, and *ACT* (according to geNorm); *ACT*, *TBP*, and *ARF1* (according to BestKeeper); *GAPC*, *TBP*, and *CYP28* (according to NormFinder); *TBP*, *GAPC*, and *UBC* (according to ΔCt method); *TBP*, *GAPC,* and *ACT* (according to RefFinder). However, the positions of the most as well as the least stably expressed genes were unaltered in the NormFinder ranking. The unstably expressed genes in the rankings were changed accordingly to the following methods: BestKeeper (*EF1A*, *SPS*, and *UBQ*), ΔCt method (*RCA*, *RPL23A*, and *EF1A*), and RefFinder (*EF1A*, *SPS*, and *RPL23A*).

### Two reference genes are required for the normalization of target genes expression results

**Figure 4:**
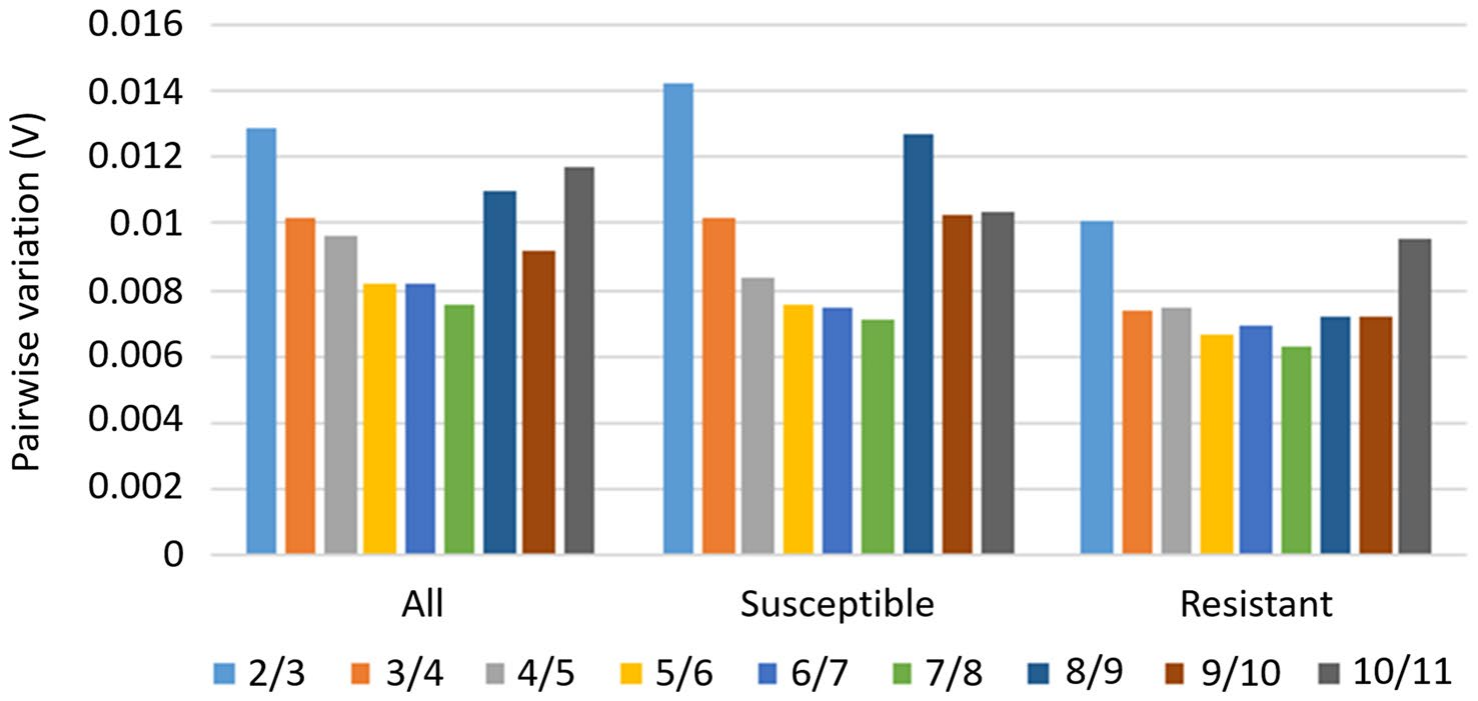
Determination of the optimal number of reference genes necessary for the accurate normalization in all tested samples, susceptible and resistant plant samples.

Pairwise variation (V_n/n+1_) between the two sequential normalization factors (NF_n_ and NF_n+1_) was calculated using geNorm. The threshold value of pairwise variation (V) below 0.15 indicates the minimum number of reference genes needed for the normalization of the target gene. In all samples collected from the plants susceptible and resistant to the herbicide, the target gene is required to be normalized using two reference genes. The V_2/3_ value was lower than 0.15, regardless of the experimental variant (Fig. 4).

### Validation of the selected reference genes revealed the constitutive *ABCC10* and *CYP89A2* up-regulation of their expression in plants resistant to pyroxsulam

ABC transporters as well as CYP450s are found to be involved in the metabolism-based NTSR to ALS inhibitors^21^. *ABCC10* was highly expressed in *Myosoton aquaticum* plants resistant to ALS inhibitor^22^, while *CYP89A2* was identified to confer metabolism‐based diclofop resistance in *Lolium rigidum*^23^. To normalize the *ABCC10* and *CYP89A2* expressions, the selected pairs of reference genes were chosen according to the efficiency corrected results. The analysis was performed using the most stably (*TBP* and *GAPC*) and the least stably (*EF1A* and *UBQ*) expressed HKGs in all samples, to show the differences in target genes expression between the pyroxsulam resistant and susceptible plants.

**Figure 5:**
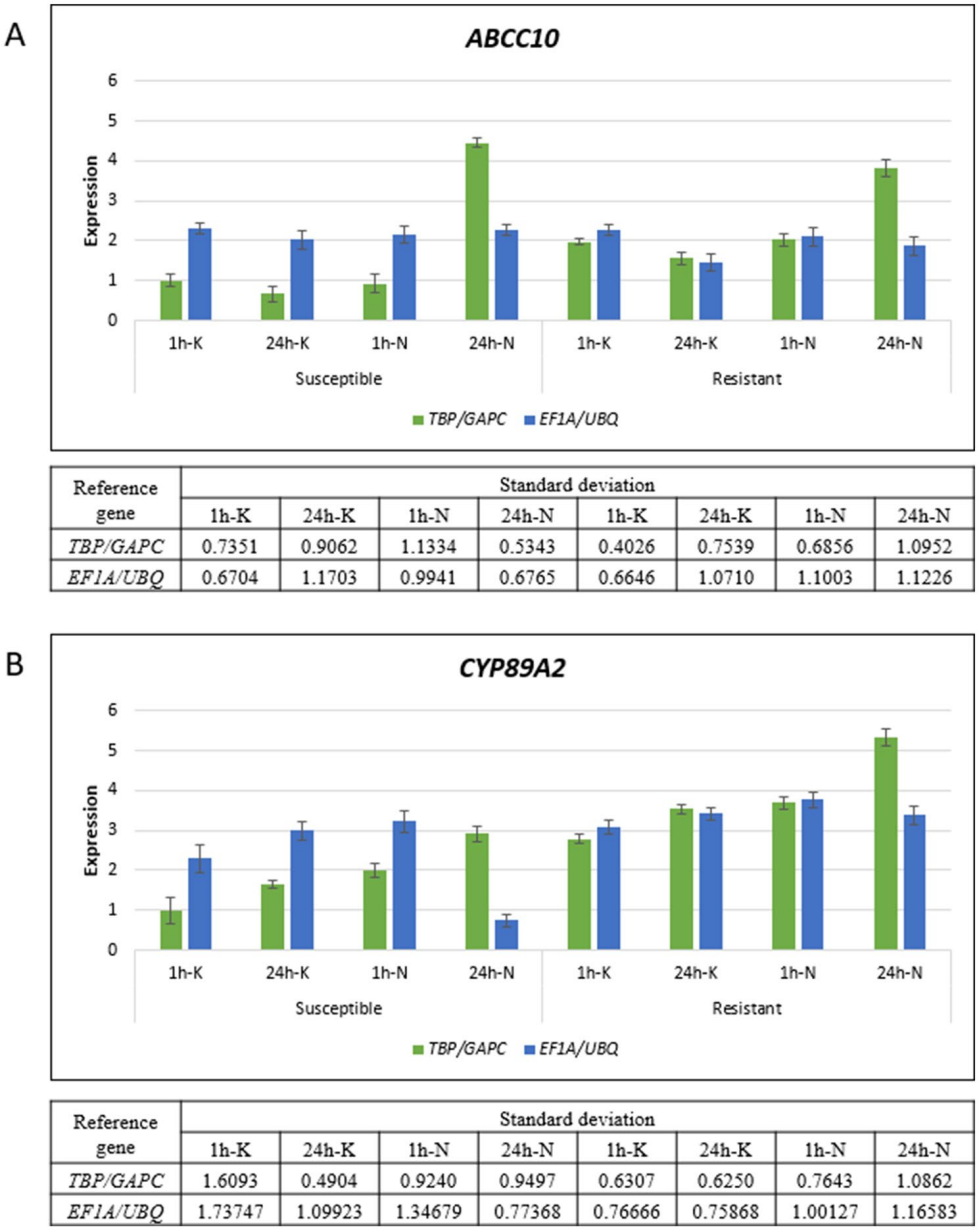
Expression of *ABCC10* (**A**) and *CYP89A2* (**B**) in *Apera spica-venti* plants non-treated or treated with pyroxsulam 1 h and 24 h after treatment with herbicide. RT-qPCR results were normalized against the most stably expressed HKGs, *TBP* and *GAPC* (green bars); and the least stably expressed, *EF1A* and *UBQ* (blue bars). Bars indicate mean expression, while the whiskers represent the standard error. *ABCC10* and *CYP89A2* expressions were set to 1.0 value in non-treated susceptible plants harvested 1 h after treatment with herbicide (1h-K), while the genes’ expression values in the remaining experimental conditions were adjusted accordingly. The tables below the charts represent standard deviation values calculated for the expression values of *ABCC10* and *CYP89A2* in each condition with the use of the most stably expressed HKGs, *TBP* and *GAPC*, and the least stably expressed, *EF1A* and *UBQ*, as normalizers. 1 h and 24 h—time after treatment; K—non-treated plants; N—plants treated with pyroxsulam.

The descriptive statistics of the results obtained with *TBP* and *GAPC* used as normalizers showed that the expression of *ABCC10* was higher in the non-treated resistant plants than in susceptible plants (Fig. 5A). Although the change in the *ABCC10* expression was higher in the pyroxsulam resistant plants 1 h after treatment with herbicide than in the susceptible ones, however, at 24 h time point, the *ABCC10* expression rapidly rose in the susceptible plants. Similarly, the change in the *CYP89A2* expression in the treated and non-treated resistant plants was higher than in the treated and non-treated susceptible ones, both at 1 h and 24 h (Fig. 5B). Moreover, the treatment with herbicide caused up-regulation of the *CYP89A2* expression.

Finally, to show how important it is to choose the most stably expressed reference genes for accurate normalization, the *ABCC10* and *CYP89A2* expression analyses were performed using the least stably expressed HKGs as normalizers, namely *EF1A* and *UBQ*. *ABCC10* was expressed at the same level in the majority of experimental conditions, except for the samples from the non-treated resistant plants harvested 24 h after treatment with herbicide, which exhibited lower *ABCC10* expression than the remaining samples (Fig. 5A). The *CYP89A2* expression analysis confirmed slight differences in the expressions between the resistant and susceptible plants, except for the samples from the susceptible plants harvested 24 h after treatment with herbicide with down-regulation in *CYP89A2* expression (Fig. 5B). Moreover, higher variance values calculated for each condition with the use of the most and the least stably expressed HKGs as normalizers, were observed for *EF1A* and *UBQ*, which indicated greater discrepancies of the obtained results within each subset of samples after normalization (Table S3). This analysis showed that the use of inappropriate normalizers changes the final results of the target gene expression analysis. Comparison of the results obtained using the most and the least stably expressed genes for normalization showed that there was not one direction of the changes in gene expression between samples. For example, the use of the least stably expressed genes for normalization led to the results indicating down-regulation of the *CYP89A2* expression in the non-treated susceptible plants harvested 24 h after treatment with herbicide, while the normalization performed with the most stably expressed genes indicated up-regulation of the *CYP89A2* expression.

## Discussion

Weed resistance to herbicides poses a serious threat to world crop production. Repetitive field applications of herbicides with the same mode of action resulted in the emergence of herbicide-resistant weed populations. The herbicide resistance may be initiated by mutations in the coding sequence of the target site of the herbicide active substance or over-production of the target enzyme. The second resistance mechanism is based, among others, on the increased metabolism and detoxication of xenobiotics. In the case of *A. spica-venti*, which was the subject of this study, TSR has been widely examined for the presence of mutations conferring resistance to ALS inhibitors^6,24,25^, while the experiments concerning NTSR mechanisms at the molecular level have been scarce. *A. spica-venti* transcriptome has been recently published^16^, however, to study the expression of the selected genes that may be associated with herbicide resistance, an internal control (reference gene) for normalization should be applied. Reference genes use is desired for the avoidance of the errors originating from the differences in RNA integrity, starting RNA quantity between samples, reverse transcription efficiency, and primer efficiency^18^. Therefore, in this study, *A. spica-venti* HKGs expression stability analysis was performed to choose the most stably expressed reference genes for the further target gene expression analyses.

According to the statistical analyses of the efficiency corrected Cq values, the best genes for expression data normalization were *TBP*, *GAPC*, and *CYP28*. The least stably expressed genes included *EF1A*, *UBQ*, and *RPL23A*. However, when the plants susceptible and resistant to ALS inhibitor were analyzed separately, the genes with the most stable expression were *CYP28*, *SPS*, and *TBP, GAPC*, respectively. *TBP* is the most stably expressed gene in *Lolium multiflorum* under salt stress and in *A. fatua* under herbicide stress^14,26^. Moreover, *TBP* was indicated as the most suitable reference gene under treatment with herbicide in *Galium aparine*^27^. *GAPDH* was also among the most stably expressed genes in herbicide-resistant grass species such as *A. myosuroides*, *A. fatua*, and *Alopecurus japonicus*^10,14,28^. *CYP28*, belonging to the cyclophilin (peptidyl-prolyl *cis–trans* isomerase) gene family was shown in our studies to be one of the most stably expressed reference genes. Cyclophilin was also used for normalization in *Cucumis melo*^29^, whereas, *SPS* was implemented as an endogenous reference gene in *Oryza sativa*^30^.

Various biotic and abiotic factors may influence housekeeping genes expression. Also, the expression stability of indicated experimentally genes might be affected in specific conditions including the plants’ developmental stage, which should be taken into account when designing an experiment using reference genes for normalization.

The validation of the most and the least stably expressed reference genes showed that the selection of the inappropriate genes in normalization might lead to the incorrect results of target gene expression analysis in the test conditions. In our study, it was revealed that the *ABCC10* expression in the pyroxsulam susceptible plants 24 h after herbicide application, varied substantially when normalization was carried out with the best and the worst reference genes. The use of the genes with unstable expression also led to the results indicating the opposite gene expression changes (from up- to down-regulation of the target gene expression). The analysis of *ABCC10,* as well as *CYP89A2* expression, showed the elevated expression of these genes after the treatment with herbicide, which implies their involvement in the plant’s reaction to the herbicide application. ABC transporters are known for their involvement in herbicide resistance through sequestration of the herbicides and their metabolites^21^. *ABCC10* was highly expressed in the plants resistant to tribenuron-methyl *M. aquaticum* and indicated to play an essential role in NTSR^22^. By contrast, the transcriptome analysis in diclofop resistant *L. rigidum* revealed the *CYP89A* expression up-regulation in the resistant, non-treated with the herbicide plants^23^. In our results, the *ABCC10* expression in the untreated *A. spica-venti* resistant to pyroxsulam plants was higher than in susceptible plants. This implies that this gene is constitutively expressed in resistant plants at a higher level than in susceptible plants, which is consistent with previous studies of *M. aquaticum*. However, *CYP89A2*, belonging to the *CYP450s* gene family participating in phase I of herbicide detoxification, also exhibits the increased expression in the non-treated resistant plants at two time points, which is also in accordance with transcriptome analysis of diclofop-resistant *L. rigidum*.

Usually, in analyses of the expression stability of HKGs by different algorithms, no efficiency corrected Cq values have been used as an input. In such situations, the assumption of 100% primers efficiency is taken, however, in practice, such a value of primer efficiency is not always achievable. Our results show that the implementation of the efficiency corrected input data changed the gene’s positions in the expression stability rankings, therefore it is important to consider the efficiency correction of the raw input data. This issue has also been brought up by another study, which indicated the necessity of performing efficiency correction of the results obtained in RT-qPCR with primers exhibiting efficiencies differing by more than 10% from the optimal 100%^15^.

## Materials and methods

### Plant material

Firstly, eleven populations of *A. spica-venti* found on Polish fields, susceptible and resistant to pyroxsulam N-(5,7-dimethoxy[1,2,4]triazolo[1,5-a]pyrimidin-2-yl)-2-methoxy4-(trifluoromethyl)pyridine-3-sulfonamide (ALS inhibitors) were examined to determine ED50. Seeds were sown in the pots placed under controlled conditions in the greenhouse. Plants at the BBCH 12–13 stage were treated with the following herbicide Nomad 75 WG (ai pyroxsulam 75 g kg^−1^, DOW AGROSCIENCES, Corteva Agriscience, Wilmington, USA) at the doses: for resistant populations: 0N, 0.5N, 1N, 2N, 4N, 8N, 16N, 32N; for susceptible populations: 0N, 1/16N, 1/8N, 1/4N, 1/2N, 1N, 2N, 4N; where N—the maximal recommended dose of the herbicide (120 g ha^−1^, i.e. 9 g ha^−1^ of active substance). Based on the obtained results, plants from the selected susceptible and resistant populations were scanned for the presence of mutations in the *ALS* sequence in order to choose populations without mutations in the ALS gene, which are known to confer the resistance.

Thereafter, the four chosen populations of *A. spica-venti* plants were used in the reference gene analysis experiment: two susceptible and two resistant to pyroxsulam (Nomad 75 WG). Plants at the BBCH 12–13 stage were treated or non-treated with the recommended dose of the herbicide (120 g ha^−1^) and harvested at 1 h and 24 h after treatment. Four biological replicates for each condition, with 2 plants per replicate, were frozen in the liquid nitrogen. All samples were stored at −80 °C until RNA extraction.

The use of plants in the present study complies with the IUCN Policy Statement on Research Involving Species at Risk of Extinction and the Convention on the Trade in Endangered Species of Wild Fauna and Flora.

### DNA isolation and *ALS* sequence analysis

Plant material was ground in a mortar using liquid nitrogen. Genomic DNA was isolated using NucleoSpin Plant II, Mini kit for DNA from plants (Mecherey-Nagel, Düren, Germany). PCR to amplify *A. spica-venti ALS* sequence was carried out in 50 µl reaction mixture containing 1× Q5 Reaction Buffer (NEB, Ipswich, MA, USA), 200 µM dNTPs, 0.5 µM forward primer (5′ ATGGCCACAGCCACGTCCA 3′), 0.5 µM reverse primer (5′ ATAAGAAAYCCTGCCATCACCKTC 3′), 200 ng of genomic DNA, and 1 U of Q5 High-Fidelity DNA Polymerase (NEB). PCR was performed in a Mastercycler^®^ nexus (Eppendorf, Hamburg, Germany) with an initial denaturation at 98 °C for 30 s, followed by 35 cycles of amplification: 10 s at 98 °C, 30 s at a 63.3 °C, and 30 s at 72 °C, with a final step of 2 min at 72 °C. The reactions’ products were separated with 1% gel electrophoresis, purified from the gel with Wizard^®^ SV Gel and PCR Clean-Up System (Promega, Madison, WI, USA), ligated to pJET1.2 plasmid using CloneJET PCR Cloning Kit (Thermo Fisher Scientific, Waltham, MA, USA), and cloned into DH10B *Escherichia coli* competent cells. The plasmids were isolated from *E. coli* cells using NucleoSpin^®^ Plasmid (Mecherey-Nagel). Three plasmids were sequenced per one plant. The presence of the insert in plasmids was confirmed by the digestion with *BglII*. DNA inserts were sequenced by Genomed (Warsaw, Poland). Sequencing data were analyzed using the BioEdit Sequence Alignment Editor 7.5.5^31^.

### RNA extraction and cDNA synthesis

Plant material was ground in a mortar using liquid nitrogen. Total RNA was extracted using 500 µl of TriReagent solution (Thermo Fisher Scientific) followed by RNA precipitation with 2-propanol. The precipitate was washed with 70% ethanol, air-dried, and suspended in nuclease-free water. The RNA concentration and purity (the 260 nm/230 nm and 260 nm/280 nm values) of each sample were estimated using a NanoDrop ND‐2000 spectrophotometer (Thermo Fisher Scientific).

Total RNA (2.5 µg) was reverse transcribed using Maxima First Strand cDNA Synthesis Kit for RT-qPCR with dsDNase (Thermo Fisher Scientific). The cDNA samples were diluted with 30 µl of nuclease-free water.

### Gene selection, primer design, and RT-qPCR

Eleven candidate reference genes were selected for the analysis: *ACT*, *ARF1*, *CYP28*, *EF1A*, *GAPC*, *RPL23A*, *RCA*, *SPS*, *TBP*, *UBC*, and *UBQ*. Moreover, 2 genes associated with NTSR were chosen for validation: encoding the ABC transporter C family member 10-like (*ABCC10*) and cytochrome P450 89A2 (*CYP89A2*). Primers sequences were designed based on the *A. spica-venti* published transcriptome (NCBI Gene Expression Omnibus GSE86989)^16^ using Primer3 software (version 0.4.0)^32^. Primers sequences list is presented in Table 1.

RT-qPCR was performed in 10 µl solution containing 1× iTaq Universal SYBR Green Supermix (Bio-Rad, Hercules, USA), 0.5 µM forward primer, 0.5 µM reverse primer, and 1 µl cDNA. The PCR program consisted of an initial incubation step at 95 °C for 3 min, followed by 40 cycles of 95 °C for 15 s, an annealing step for 30 s (temperatures listed in Table 1), and 72 °C for 30 s. The dissociation curves were generated from 60 to 95 °C. The standard curve for each gene was made on the basis of n-fold dilutions of cDNA. Reactions were performed in triplicate (technical replicates) in QuantStudio5 (Thermo Fisher Scientific). However, to optimize reactions’ conditions for each pair of primers, firstly RT-qPCR with temperature gradient at the annealing step was performed. To confirm the estimated size of the amplicons, the reactions’ products were resolved on a 2% agarose gel, purified from the gel with Wizard^®^ SV Gel and PCR Clean-Up System (Promega), ligated to pJET1.2 plasmid using CloneJET PCR Cloning Kit (Thermo Fisher Scientific), and cloned into DH10B *Escherichia coli* competent cells. The plasmids were isolated from *E. coli* cells using NucleoSpin^®^ Plasmid (Mecherey-Nagel), followed by the confirmation of insert presence in plasmids by the digestion with *BglII*. DNA inserts were sequenced by Genomed (Warsaw, Poland). Sequencing data were analyzed using the BioEdit Sequence Alignment Editor 7.5.5^31^. The obtained sequences were compared with sequences of the corresponding transcript in *A. spica-venti* transcriptome and submitted to the GenBank database. To confirm the similarity of the sequences of products amplified by the designed primers targeting HKGs encoding genes to the selected genes, they were blasted to the following databases: NCBI blastn and UniProt blastp. Moreover, the sequences were blasted against a model plant species *A. thaliana* TAIR database (Araport11 Proteins) using the website BLASTP search (version 2.9.0+, https://www.arabidopsis.org/Blast/index.jsp) and against the closest to *A. spica-venti* model plant species *B. distachyon* protein sequences (v3.2)^33^ downloaded from Phytozome^34^ using OmicsBox^35^.

### Data analysis and validation

Distribution of candidate reference genes Cq, efficiency correction of Cq data as well as the normalization of the expression of *CYP89A2* and *ABCC10* using the most and the least stably expressed genes were performed with GenEx (version 6.1.1.550, Multid Analyses AB, Göteborg, Sweden). The expression of *CYP89A2* and *ABCC10* was calculated using descriptive statistics at the confidence level of 95%.

To determine the expression stability of the reference gene candidates for *A. spica-venti*, the analyses were carried out using four independent bioinformatics algorithms: geNorm^17^ and BestKepeer^18^ softwares, performed in the integrated development environment (IDE) of R (RStudio), NormFinder software^19^ in GenEx (version 6.1.1.550), and ΔCt method^20^ recalculated in Microsoft Excel application (Microsoft Office 2018). To gain a comprehensive list of genes expression stability by calculating the geometric mean, RefFinder software (https://www.heartcure.com.au/reffinder) was implemented. The analyses were performed using pooled Cq data from susceptible and resistant samples together, pooled Cq values for the susceptible samples only, and separately for the resistant samples only.

To conduct genes expression stability analysis in the R software environment, the data from RT-qPCR analysis (non-corrected or efficiency corrected Cq values) were loaded to R software (version 3.6.2)^36^. To conduct expression stability analysis of the selected candidate genes, two different packages were installed: “NormqPCR” (version 1.7.1)^37^ (geNorm) and “ctrlGene” (version 1.0.1)^38^ (BestKeeper). The first one calculates the average expression stability values (M) of the analyzed genes during stepwise exclusion of the least stably expressed genes in each round until the most stably expressed candidates remain. The second one calculates descriptive statistics from Cq data and pairwise correlation between all analyzed genes. The results of gene expression stability analysis from BestKeeper software are calculated given the standard deviation (SD) of Cq values between all analyzed genes, where the lowest SD score represents the gene with the most stable expression in ranking index^37^. In order to determine the minimum required number of reference genes for the analysis, the algorithm in geNorm calculates the pairwise variation (V_n/n+1_) between the two sequential normalization factors (NF_n_ and NF_n+1_) with the cut-off threshold of 0.15^38^.

The NormFinder software calculates a global average expression of all genes in the model studied, to which the individual genes are compared. Next, the SD is estimated for each candidate gene and separates the variation into intragroup and intergroup contribution^19^. The ΔCt method calculates the expression stability of individual genes by comparing the relative expression of two reference genes pairwise. At the first step, the delta Cq is estimated between each pair of tested genes with the SD values of the obtained results. Then, a fixed base index of SD values for each tested-gene with its all possible pair is generated. The expression stability of candidate genes is ranked according to the mean values of SD recalculated for each index^20^.

### Consent for publication

All authors have consented to this publication.

## Supplementary Information

Supplementary Tables.

## Data Availability

All data generated or analyzed during this study are included in this published article.
