# Supplementary material for: The analysis of reference genes expression stability in susceptible and resistant Apera spica-venti populations under herbicide treatment
Source: Sci Rep. 2021 Nov 12;11:22145. doi: 10.1038/s41598-021-01615-6 (PMC8589970; doi:10.1038/s41598-021-01615-6)
Supplement: Supplementary file 1 — Supplementary Tables. [file 41598_2021_1615_MOESM1_ESM.docx]

**The analysis of reference genes expression stability in susceptible and resistant *Apera spica-venti* populations under herbicide treatment**

Barbara Wrzesińska^1^, Karolina Kościelniak^1^, Patryk Frąckowiak^1^, Tadeusz Praczyk^2^, Aleksandra Obrępalska-Stęplowska^1^*

^1^ Institute of Plant Protection – National Research Institute, Department of Molecular Biology and Biotechnology, Władysława Węgorka 20, 60-318 Poznań, Poland

^2^ Institute of Plant Protection – National Research Institute, Department of Weed Science and Plant Protection Techniques, Władysława Węgorka 20, 60-318 Poznań, Poland

* Corresponding author: Aleksandra Obrępalska-Stęplowska. tel: (+48) 61 864 9145; e-mail: olaob@o2.pl or ao.steplowska@iorpib.poznan.pl

**SUPPELEMENTARY FILES**

**Table S1.** ED50 determination for eleven *Apera spica-venti* populations treated with Nomad 75 WG (active ingredient (ai) pyroxsulam 75 g kg^-1^, DOW AGROSCIENCES, Corteva Agriscience, Wilmington, USA) at the doses: for resistant populations: 0N, 0,5N, 1N, 2N, 4N, 8N, 16N, 32N; for susceptible populations: 0N, 1/16N, 1/8N, 1/4N, 1/2N, 1N, 2N, 4N; where N – the maximal recommended dose of the herbicide (120 g ha^−1^, i.e. 9 g ha^-1^ of ai). S – sensitive population, R – resistant population. Names in bold represent populations subjected to housekeeping gene stability analysis

| Population name | ED50 [ai g h^-1^] |
| --- | --- |
| **S1** | **<0.5625** |
| **S2** | **<0.5625** |
| **R1** | **17.45** |
| **R2** | **>288** |
| R3 | >288 |
| R4 | 10.46 |
| R5 | >288 |
| R6 | 66.82 |
| R7 | >288 |
| R8 | 76.31 |
| R9 | 134.76 |

**Table S2.** Blast searches of the obtained in RT-qPCR products’ sequences of *A. spica-venti* HKGs against NCBI and UniProt databases, as well as, the search was done against model plant species *Brachypodium distachyon* and *Arabidopsis thaliana*. Accessions with the highest scores are presented

| Gene | NCBI blastn | UniProt blast | *Brachypodium distachyon* - blastp | *Arabidopsis thaliana* – blastp |
| --- | --- | --- | --- | --- |
| ACT | AY014279.1 *Lolium perenne* actin gene, partial cds | P02582 Actin-1 (*Zea mays*) | Bradi2g56000.1 PTHR11937:SF205 - ACTIN // SUBFAMILY NOT NAMED | AT5G09810.1 AtACT7,ACT7 \| actin 7 |
| ARF1 | XM_040398420.1 PREDICTED: *Aegilops tauschii* subsp. *strangulata* ADP-ribosylation factor-like protein 1 (LOC109747793), transcript variant X2, mRNA | M7ZU18 ADP-ribosylation factor-like protein 1 (*Triticum urartu*) | Bradi1g21210.3 K07952 - ADP-ribosylation factor related protein 1 (ARFRP1) | AT5G52210.2 ATGB1,ATARLB1,GB1 \| GTP-binding protein 1 |
| CYP28 | XM_010236252.3 PREDICTED: *Brachypodium distachyon* peptidyl-prolyl cis-trans isomerase CYP28, chloroplastic (LOC100846319), transcript variant X2, mRNA | A0A5J9W3B7 PPIase cyclophilin-type domain-containing protein (*Eragrostis curvula*) | Bradi3g19920.4 PTHR11071:SF286 - PEPTIDYL-PROLYL CIS-TRANS ISOMERASE // SUBFAMILY NOT NAMED | AT5G35100.1 Cyclophilin-like peptidyl-prolyl cis-trans isomerase family protein |
| EF1A | MH450182.1 *Alopecurus aequalis* elongation factor 1 mRNA, complete cds | Q40034 Elongation factor 1-alpha (*Hordeum vulgare*) | Bradi0012s00100.1 K03231 - elongation factor 1-alpha (EEF1A) | AT5G60390.3 EF1alpha \| GTP binding Elongation factor Tu family protein |
| GAPC | XM_003573269.4 PREDICTED: *Brachypodium distachyon* glyceraldehyde-3-phosphate dehydrogenase 1, cytosolic (LOC100843479), mRNA | P26517 Glyceraldehyde-3-phosphate dehydrogenase 1, cytosolic (*Hordeum vulgare*) | Bradi3g14120.4 PTHR10836:SF58 - GLYCERALDEHYDE 3-PHOSPHATE DEHYDROGENASE // SUBFAMILY NOT NAMED | AT3G04120.1 GAPC1, GAPC, GAPC-1 \| GLYCERALDEHYDE-3-PHOSPHATE DEHYDROGENASE C SUBUNIT, GLYCERALDEHYDE-3-PHOSPHATE DEHYDROGENASE C SUBUNIT 1 |
| RPL23A | XM_010241777.3  PREDICTED: *Brachypodium distachyon* 60S ribosomal protein L23A (LOC100823942), transcript variant X2, mRNA | M8A553 60S ribosomal protein L23a (*Triticum urartu*) | Bradi5g14750.2 K02893 - large subunit ribosomal protein L23Ae (RP-L23Ae, RPL23A) | AT3G55280.3 RPL23A2,RPL23AB \| RIBOSOMAL PROTEIN L23A2 |
| RCA | AY312573.1 *Deschampsia antarctica* Rubisco activase alpha form precursor (RCA1) mRNA, complete cds; nuclear gene for chloroplast product | Q7X9A1 Rubisco activase beta form (*Deschampsia antarctica*) | Bradi4g09125.22 KOG0651 - 26S proteasome regulatory complex, ATPase RPT4 | AT2G39730.1 RCA \| rubisco activase |
| SPS | XM_037554986.1 PREDICTED: *Triticum dicoccoides* probable sucrose-phosphate synthase 4 (LOC119274305), mRNA | A0A446N0A6 Sucrose-phosphate synthase (*Triticum turgidum* subsp. *durum*) | Bradi3g20120.5 PTHR12526:SF334 - GLYCOSYLTRANSFERASE // SUBFAMILY NOT NAMED \| Putative sucrose phosphate synthase. Glycosyltransferase in family GT4 | AT5G11110.1 SPSA2,KNS2,SPS1,ATSPS2F,SPS2F \| sucrose phosphate synthase 1, sucrose phosphate synthase 2F, sucrose-phosphate synthase A2 |
| TBP | XM_037566655.1 PREDICTED: *Triticum dicoccoides* TATA-box-binding protein 2 (LOC119287135), transcript variant X2, mRNA | P93348 TATA-box-binding protein (*Nicotiana tabacum*) | Bradi4g01150.6 transcription initiation factor TFIID TATA-box-binding protein (TBP, tbp) | AT1G55520.3 TBP2,ATTBP2 \| TATA BINDING PROTEIN 2 |
| UBC | XM_020305419.2 PREDICTED: *Aegilops tauschii* subsp. *strangulata* probable ubiquitin-conjugating enzyme E2 24 (LOC109746297), mRNA | A0A3B6A2D9 UBC core domain-containing protein (*Triticum aestivum*) | Bradi2g16960.9 PTHR24067:SF97 - UBIQUITIN-CONJUGATING ENZYME E2 // UBIQUITIN-CONJUGATING ENZYME E2 24-RELATED | AT2G16920.2 PFU2, UBC23 \| ubiquitin-conjugating enzyme 23 |
| UBQ | XM_003568817.4 PREDICTED: *Brachypodium distachyon* ubiquitin-40S ribosomal protein S27a (LOC100831343), mRNA | G3DQF2 Ubiquitin (*Lolium perenne*) | Bradi2g34750.1 K02977 - small subunit ribosomal protein S27Ae (RP-S27Ae, RPS27A) \| ubiquitin-protein ligase activity (Blast2GO) | AT4G05320.4 UBQ10,UBI10 \| ubiquitin 10, polyubiquitin 10 |
| ABCC10 | KP852211.1 *Beckmannia syzigachne* ABC transporter C family member 10-like mRNA, complete sequence | M8BT23 ABC transporter C family member 10 (*Aegilops tauschii*) | Bradi1g48708.1 PTHR24223:SF192 - FAMILY NOT NAMED // ABC TRANSPORTER C FAMILY MEMBER 10 | AT1G19430.2 S-adenosyl-L-methionine-dependent methyltransferases superfamily protein |
| CYP89A2 | XM_003574128.*4* PREDICTED: *Brachypodium distachyon* cytochrome P450 89A2 (LOC100824412), mRNA | R7W153 Cytochrome P450 89A2 (*Aegilops tauschii*) | Bradi3g30510.1 KOG0156 - Cytochrome P450 CYP2 subfamily | AT1G64940.1 CYP89A6 \| "cytochrome P450, family 87, subfamily A, polypeptide 6 |

**Table S3.** Variance values calculated for the expression values of *ABCC10* and *CYP89A2* in each condition with the use of the most stably expressed HKGs, *TBP* and *GAPC*, and the least stably expressed ones, *EF1A* and *UBQ*, as normalizers. 1 h and 24h – time after treatment; K – non-treated plants; N – plants treated with pyroxsulam

| Reference gene | Condition | | | | | | | |
| --- | --- | --- | --- | --- | --- | --- | --- | --- |
|  | Sensitive populations | | | | Resistant populations | | | |
|  | 1h-K | 24h-K | 1h-N | 24h-N | 1h-K | 24h-K | 1h-N | 24h-N |
| *ABCC10* | | | | | | | | |
| *TBP*/*GAPC* | 0.5404 | 0.8212 | 1.2846 | 0.2855 | 0.1621 | 0.5684 | 0.4700 | 1.1995 |
| *EF1A*/*UBQ* | 0.4494 | 1.3697 | 0.9881 | 0.4577 | 0.4417 | 1.1471 | 1.2107 | 1.2603 |
| *CYP89A2* | | | | | | | | |
| *TBP*/*GAPC* | 2.5898 | 0.2405 | 0.8538 | 0.9019 | 0.3978 | 0.3906 | 0.5842 | 1.1798 |
| *EF1A*/*UBQ* | 3.0188 | 1.2083 | 1.8138 | 0.5986 | 0.5878 | 0.5756 | 1.0025 | 1.3592 |
